# Sequential Localizing and Mapping: A Navigation Strategy via Enhanced Subsumption Architecture

**DOI:** 10.3390/s20174815

**Published:** 2020-08-26

**Authors:** Kamal M. Othman, Ahmad B. Rad

**Affiliations:** Autonomous and Intelligent Systems Laboratory, School of Mechatronic Systems Engineering Simon Fraser University, Surrey, BC V3T 0A3, Canada; kamal_othman_2@sfu.ca

**Keywords:** social robots, robotics navigation, subsumption architecture, reinforcement learning, SLAM

## Abstract

In this paper, we present a navigation strategy exclusively designed for social robots with limited sensors for applications in homes. The overall system integrates a reactive design based on subsumption architecture and a knowledge system with learning capabilities. The component of the system includes several modules, such as doorway detection and room localization via convolutional neural network (CNN), avoiding obstacles via reinforcement learning, passing the doorway via Canny edge’s detection, building an abstract map called a Directional Semantic Topological Map (DST-Map) within the knowledge system, and other predefined layers within the subsumption architecture. The individual modules and the overall system are evaluated in a virtual environment using Webots simulator.

## 1. Introduction

Social robots are referred to as a special family of autonomous and intelligent robots that are predominantly designed to interact and communicate with humans or other robots (agents) within a collaborative environment. Embodiment is an essential characteristic of this class of robots. As such, avatars and virtual agents are generally excluded. Social robots are designed for a variety of tasks in a collaborative or service setting and could be deployed in homes (to do household chores, act as a companion to children and seniors, or serve as a butler, etc.), hospitals (as a nurse, administrative assistant, etc.), schools (as a teacher), libraries (as a librarian), museums (guides, etc.), to name a few. Other key features of such robots are their ability to recognize people, objects, communicate through voice, and respond to various human emotions. Social robots are designed in all sizes and shapes for a variety of applications but most importantly, they are designed to be acceptable to humans. Depending on its ultimate application, a social robot can be designed as a pet-like, e.g., AIBO [1], or a humanoid, e.g., Nao [2], or a wheeled robot, e.g., Pepper [3], or unmovable robot, e.g., Kasper [4].

Implicit but an indispensable feature of a social robot is its ability to seamlessly move around in the environment for which it is expected to function. Indeed, a social robot cannot realistically perform its dedicated tasks, if it is immobile. It is, thus, sensible to suggest that such skills can hardly be isolated from the crucial ability to explore, navigate, or perceive information in the way humans do. Hence, designing a robotic navigation system with limited sensors that is capable of exploring an indoor environment while the robot concurrently updates its pose and generates/updates its map is an active research area in robotics. This problem has been studied within the context of probabilistic and behavioristic paradigms, respectively. Whereas the former has been extensively studied with successful implementations; the interest in the latter, after a dormant period, has been revived in the last decade through advancements of deep learning and problem-solving algorithms inspired by the natural world.

In this paper, we report a methodology within the general behavioristic architecture (Sense-Act system). It could be argued that this strategy is more challenging than a purely deliberative design that depends on prior planning and an accurate map. Additionally, the conventional behavioristic design often acts blindly and does not explicitly include a learning module that is essential for the robot (agent) to function in a purposeful manner. To circumvent this shortcoming, we propose a behavioristic robotic system that not only guides a social robot to explore a home environment with only modest prior knowledge but also builds a local map and registers its location within that map. We coin the term Sequential Localization and Mapping (SeqLAM)—not to be confused with the widely popular probabilistic Simultaneous Localization and Mapping (SLAM) algorithm [5]. The proposed system consists of several layers based on Brook’s subsumption architecture [6], in which each layer is responsible for a specific task. The goal of the system is to enable a social robot to navigate an indoor environment safely and purposively, to avoid obstacles, to go to a specific location within the environment, and to build a map for future visits. The purposive capability of the system is manifested through an integrated knowledge-based system that provides the robot’s location and builds an abstract map sequentially. In contrast to SLAM, which employs an explicit incremental coordination-based location (pose), a zone-based location is identified in SeqLAM. As for the abstract map, we employ a Directional Semantic Topological Map (DST-Map) that takes advantage of different zones and the spatial relationships between the zones.

We will go through the detailed components of the proposed system in this paper. However, we first need to set the scene and provide context for the design. Here, the term indoor environment is specific and is reserved for human habitats, i.e., an apartment. As it will be shown later, the zones are referred to as five classes (bathroom, bedroom, dining room, kitchen, and living room) that are generally present in a home. The proposed design can be readily extended to homes with more than one of each class (two bedrooms, etc.) and other indoor settings such as a hospital, a library, an office, etc. To demonstrate its performance, the system is implemented and tested virtually on the Nao humanoid robot within a small apartment.

The rest of the paper is organized as follows: in Section 2, we present related studies of performing exploration and SeqLAM with a focus on reactive systems integrated with knowledge systems. This section will also provide an explanation of the Subsumption architecture as well as the approach of reinforcement learning (RL), which are adopted in this study. Then, the proposed system and the design of each module will be explained in Section 3. We will then include and discuss experiments for individual modules and the overall system in Section 4. The paper concludes with a summary and further remarks on the overall system in Section 5.

## 2. Background and Related Research

Consider a scenario whereby a social robot is required to safely explore and learn in a new environment; in the context of this study, a new apartment. In robotics literature, this task is broadly referred to as robotics exploration, which includes wandering in an unknown environment with the purpose of gaining information of that environment (building a map) using mainly exteroceptive sensors. This problem has been addressed by different approaches, including but not limited to geometric or frontier-based methods (maintaining boundaries) [7,8], information-theoretic or probabilistic methods (minimizing uncertainty) [9,10,11], and data-driven or learning-based methods (predicting a map via trained network on a dataset of partial maps) [12,13]. Simultaneous Localization and Mapping (SLAM) is widely considered as the main strategy to accomplish this task. SLAM is defined as a robotics navigation process through which a robot builds a map of an unknown environment while simultaneously estimating its pose within the created map. SLAM is essentially a probability-based technique as it deals with an inherently uncertain and noisy measurements. Several probabilistic techniques have been employed in a SLAM algorithm, including but not limited to, Kalman Filter (KF), Particle Filter (PF), or Expectation-Maximization (EM) [14,15].

In parallel, the bio-inspired SLAM approaches have also been studied. Such algorithms are distinguished by designs that are motivated by nature to address the robotic navigation problem via developing and validating a biological design, e.g., RatSLAM and BatSLAM. The RatSLAM [16,17] is a visual-based structure that was inspired by the connection of different types of cells in the rat’s hippocampus. The structure is a fixed-weights network for pose cells. The BatSLAM [18], on the other hand, has basically the same structure as the RatSLAM, however, it is based on biomimetic sonars instead of a monocular camera.

Designing a high-level control architecture is also important in any robotic navigation task, including exploration strategies and SLAM. There are several types of robotic architectures suggested by robotics researchers [19]: deliberative system that is also known as SPA (Sense-Plan-Act), reactive robotic system that is known as SA (Sense-Act) system, and the hybrid system that integrates the two previous systems. Since a map does not generally exist a priori, behavior-based architectures, i.e., reactive method, can be considered as an alternative solution. One of the key behavior-based architectures is the ubiquitous subsumption architecture that was proposed by Brook [6]. The main characteristic of this methodology is to eliminate the plan function from the navigation system while the system decouples the sense and the act functions in the form of distinct behaviors. The subsumption architecture falls under the behavioristic psychology that generally claims that a behavior is triggered by the environment, as opposed to cognitive psychology that argues mental (internal) representations play a causal role in behavior [20]. As such, the original architecture did not explicitly learn from experience. We argue that while adhering to the overall architecture, it can be enhanced by embedded learning that incorporates learned knowledge in decision making during navigation. Accordingly, in this paper, we focus on studying these issues by developing an integrated indoor navigation system via an enhanced subsumption architecture.

This perspective has been studied by other researchers, albeit quite different from the design herein. One of the early studies was by Arkin [21] who presented a hybrid architecture that combined two independent levels of planning and action, which were based on the potential field method [22]. He also discussed the definition and the importance of maintaining knowledge within a robotic system in his celebrated textbook “Behavior-Based Robotics” [23]. Similarly, the same concept was adopted in several studies with different methods. In [24], a hybrid deliberative-reactive architecture was proposed in which behaviors of the reactive system were designed based on fuzzy logic, while the path planning was addressed based on a prior given map of a static environment. In [25], the authors presented a hybrid navigation system (deliberative and reactive) with incomplete knowledge, i.e., known positions of some static obstacles. The deliberative part was designed based on a binary gird map with the A* algorithm to generate a global path, while the reactive part was designed based on the DH-bug algorithm. It was tested via simulation studies on a Pioneer robot with laser and sonar sensors. Furthermore, the studies in [26] and [27] suggested a cognitive method for planning level and a learner method for the reactive level. The authors used a minefield simulator to evaluate the performance of the BDI-FALCON hybrid system for an autonomous vehicle with five sonar sensors. The FALCON was a low-level reinforcement learner, while the BDI (Belief-Desire-Intention) is the high-level planner using prior data. The FALCON system took the action when there was no available plan. Once the plan was created, the FALCON system was suppressed and the action was executed by the BDI system. These aforementioned studies demonstrated designs based on a deliberative system that generally required an accurate map which they could use independently within the behavioristic domain.

Alternatively, Mataric [28] designed an architecture that integrated a map representation with a reactive system, which was tested on a mobile robot equipped with a ring of sonars and a compass within an office environment. The main three levels of the architecture were: subsumption for navigation, wall detection (left, right corridor), and map building (topological) that consisted of nodes with four attributes, including metric information. In [29], Case-Based Reasoning (CBR) was suggested to address the knowledge for a reactive system within a static environment. The concept of CBR is to solve a current problem by retrieving past experiences. The created cases in this study, i.e., pairs of sense-act, consisted of sonars readings, robot direction, and goal direction, while the output was the heading direction. Conversely, the study in [30] focused only on the learning ability within a reactive system and suggested a combination of two independent systems: a reactive system based on potential field method, and a learning system based on Reinforcement Learning (RL). The RL component was integrated to coordinate layers in the reactive system that enhanced the robot’s movement toward the goal within an unknown non-convex environment. Additionally, the reactive navigation was addressed in simulation trials [31] by designing two simple behaviors (avoid obstacle and go to goal) within actor-critic architecture for a wheeled robot in a static environment. The reactive system was combined with a trajectory generator and a tracking control system in a hierarchical theme. In addition, planning a trajectory for reactive navigation was solved in [32], based on the law of electromagnetism that leads the arm robot to a desired predefined position while avoiding unknown obstacles. From these studies, the static environments are the main assumption of the reactive systems by combining different types of knowledge/learning or focusing on the trajectory problem based on a partially known environment.

Within the probabilistic approaches, studies that integrated SLAM with various robotic control systems have also been reported. Visual SLAM was used for a deliberative control system in [33]. The authors presented a theoretical control architecture for outdoor navigation using only a single camera. The system started with two visual modules: structure from motion and visual SLAM. The first module took a sequence of 2D images of the same scene from different viewpoints to get depth information, i.e., reconstruct a 3D map. The depth information was passed to “Avoid Obstacle” behavior. The other module performed SLAM via images followed by a path planner that provided waypoints to be used for “Go to Goal” behavior. The outputs of these two behaviors were fused as a final command control. In contrast, the authors in [34] combined the probabilistic SLAM module with behavior-based motion module within a control system to address the exploration problem for an aerial robot. The SLAM module provided the estimated robot position, whereas the motion module provided the desired position. Both values were passed to a space-state model (low-level) controller to minimize the error. In addition, the authors in [35] aimed to improve the performance of a reactive system by integrating probabilistic SLAM to address a biohazard search mission in an unknown environment. The SLAM algorithm was a probabilistic approach called GMapping that built a metric grid map based on particle filter and localized the robot position within the map. Whereas, the reactive system consisted of a collection of behaviors that were connected based on the form of a finite state machine (FSM) or a finite state automaton (FSA). Behaviors in the reactive system were triggered by the spatial memory of SLAM instead of the stimulus inputs. All their experiments were executed on a Pioneer 3 wheeled robot with laser for performing SLAM and camera for detecting the target within a limited and static environment. As we noticed from these studies, probabilistic SLAM for a static environment, which is mainly for uncertainty issue, was added to the system. However, the uncertainty in position and mapping, and the static environment are not viable assumptions in real settings.

In contrast to the aforementioned studies, we focus on designing a robotic exploration system that integrates learning and knowledge capability to a reactive system for social robots with limited sensors. Thus, the assumption of static environments is relaxed. Besides, the knowledge system will be designed for addressing localization and mapping in an abstract manner. Accordingly, we design a behavioristic system based on subsumption architecture. We also design a knowledge system that builds a Directional Semantic Topological Map (DST-Map) incrementally, which is accessed by all layers in the subsumption system. Therefore, behaviors in layers can be triggered based on the direct stimulus from sensors as well as the gained information in the DST-Map. Thus, the proposed system will build a map and sequentially localize the robot within the abstracted created map during the exploration process. We refer to it as a sequential localization and mapping (SeqLAM) that does not localize incrementally but identifies itself in a predefined zone. In addition, an appropriate reinforcement learning module is designed for adaptive behavior while exploring using only two sonars. All designed modules and the overall system are implemented and tested on the Nao humanoid robot within a house environment using Webots simulator [36].

With the proposed behavioristic system, associated issues to the exploration and SLAM with classical techniques will be addressed or improved. For example, the semantic information within the DST-Map helps SLAM via recognizing the scene and classifying the type of the room [37,38] which gives a meaningful localization and mapping. Consequently, other robotic applications will be improved because of the meaningful task such as the interaction between humans and robots or switching the planning function from a path issue to a task issue, e.g., the robot needs to go to the bedroom. Furthermore, The DST-Map implicitly addresses the associated issues to SLAM: high dimensionality and data association [14,15]. The former issue is addressed within our proposed system as the nodes in the DST-Map represents the high-level locations instead of objects or free space locations. Whereas, the latter issue is addressed through matching the connection between nodes within the created DST-Map. If there are multiple connected rooms, e.g., two different bedrooms are connected to two different bathrooms, then it is flexible to add a new module to the system for image matching. In contrast to most SLAM studies that focused on addressing localization and mapping while controlling the robot was executed manually, this work addresses the control part via a collection of behaviors that is useful during any interaction between humans and robots.

### 2.1. Subsumption Architecture

**Figure 1:**
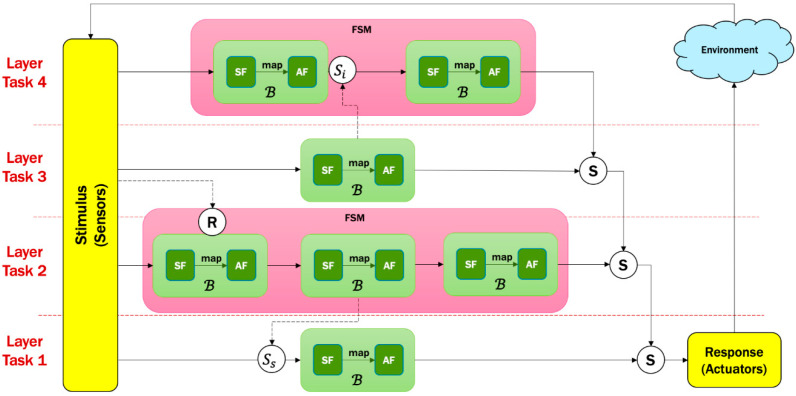
An illustration of subsumption architecture with layers and possible components. (𝓑: Behavior, **SF**: Sense Function, **AF**: Act Function, **FSM**: Finite State Machine,$\;S_{i}$: Inhibitor, $S_{s}$: Suppressor, **R**: Releaser, **S**: Subsumed layer by the higher layer).

Subsumption architecture is the first purely reactive robotic control system, which was introduced by Brook in his early work on the behavior-based system [6]. He argued against explicit modelling of the world and notably stated that “the world is its own best model” [39]. This system consists of a set of layers, as shown in Figure 1, in which each layer is responsible for a specific task. These layers are built in parallel where the higher layers have more dominance than the lower ones when they are triggered concurrently, i.e., competition coordination. Each layer is decomposed into basic behaviors or modules. A module is considered as a pre-wired reflexive behavior that connects a sense function with an act function in order to perform a specific behavior. Each behavior can be created, tested, and debugged individually.

In the robotics navigation context, the system decomposes the navigation problem into vertical task-achieving behaviors, such as avoid, wander, explore … etc. Layers are added to the system incrementally, and they are built and activated in parallel, but operated asynchronously. Let us consider that level 0, e.g., “move around” behavior, is designed, implemented and debugged. Then, level 1 is added, e.g., “obstacle avoidance” behavior, with keeping the function of level 0. Therefore, the robot can move and detect obstacles to be avoided. The main feature of subsumption is that the higher layer can subsume the control of the lower layer, which is the origin of the name, when behaviors are triggered concurrently. Consequently, the higher layer only controls the robot to the overall goal or destination at a specific time. Since interaction between layers might become complicated with complex environments and tasks, the goal in subsumption design is to keep the connection between layers as short as possible, which is more effective for modularity. In short, behaviors in the subsumption system are coordinated and executed such that the robot can interact with the environment and select the best behavior in a sequential manner until the task is completed with no plan for future or any memory of past knowledge.

Each behavior is designed by mapping a stimulus (input sensory data) into a response behavior (actuator output) through connecting its own sense and act functions. In addition, a behavior can be modified by its respective inhibitor, suppressor, or releaser conditional functions, as shown in Figure 1. An inhibitor function ($S_{I}$) is designed to inhibit a behavior from controlling the robot even if the sensor data is available. A suppressor function ($S_{S}$), on the other hand, suppresses the corresponding behavior output, and consequently, no output response is generated. A releaser is similar to a switch that turns the module on/off based on a particular sensory input. In the case of sequence behaviors, such as finding a refrigerator, opening the door, finding a can, gripping a can, moving gripper out of the refrigerator, and closing the door, this type of task can be accomplished by a Finite State Machine (FSM). This implies that instead of letting one behavior trigger the next behavior, each behavior in this sequence can be activated through the environment. For example, if the state of the refrigerator door is “open”, then “find a can” behavior will be activated, or if the state of the gripper is “closed”, then “moving gripper out of refrigerator” will be activated, and so on. Hence, the design and the coordination between behaviors essentially depend on the application or the main task.

Brooks [39] configured a number of MIT’s robots with the subsumption system, Toto, Genphis, and Seymour. Subsumption architecture has several advantages including low computation as there is no need for a model and no plan modules; consequently, it is fast. However, the pure reactive behaviors in subsumption are not straightforward nor reliable for complex tasks in complex environments as there is no clear final goal within the system, i.e., no plan. In addition, there is no explicit learning, no memory, and no goal-directed motivation [40]. Although some researchers attempted addressing these shortcomings by integrating different algorithms of soft computing with subsumption such as fuzzy logic [41], genetic algorithm [42], or neural network [43], further research on subsumption lost its momentum; particularly due to the emergence and success of probabilistic robotics. Integrating learning capability with behaviors can be categorized into two main types: learning coordinating behaviors and learning new behaviors [44]. It is also sporadic regarding employing reinforcement learning (RL) within behaviors, specifically in subsumption architecture. Two early studies were reported by Mahadevan and Connell [45] and Mataric [46] using RL. In [45], RL was combined with statistical clustering and hamming distance in a box-pushing task within subsumption, whereas in [46] RL was applied in a multi-robot domain. Recently, RL has been used with a behavior-based system in order to learn how to perform new tasks, and its performance has been compared with subsumption in [47]. The design consists of two combined phases. First is the imitation phase, which is carried out by using a self-organizing decision tree to emulate the behavior of a skilled operator. Second is the composition phase, which is accomplished by using Q- learning to combine all behaviors and assign learned weights. Then, the outputs of all behaviors from phase one and the learned weights from phase two are fused to perform weighted action. In addition, Q-learning was applied, and its performance was compared within two different behavior-based systems: subsumption and motor schema. They were tested on the Lego NXT robot for avoiding obstacles [48].

With the advent of machine learning and particularly deep learning in the last two decades, new opportunities arise to enhance the subsumption architecture and alleviate its shortcomings. In this paper, we propose a subsumption-based system that is integrated with a knowledge system. We demonstrate its performance to address the open problem of exploration in structured indoor settings. The new system has learning capabilities for addressing specific perceptual and action problems.

### 2.2. Reinforcement Learning (RL)

**Figure 2:**
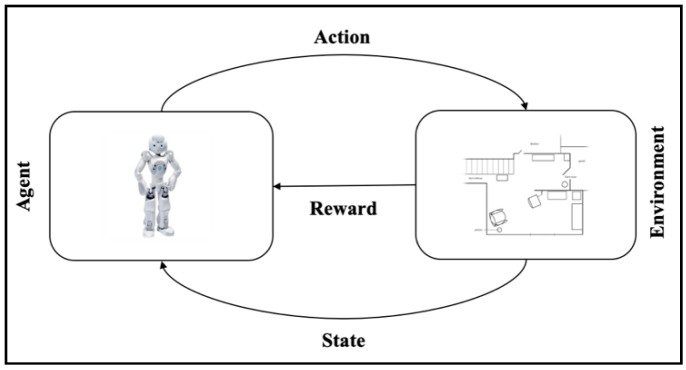
The concept of the RL approach.

The revival of reinforcement learning (RL) can arguably be credited to Richard S. Sutton, who is considered as the “father” of RL in the early 1980s [49]. RL is a class of machine learning algorithms that was inspired by learning theory in animal behaviors [50,51]. The main characteristic of RL is that the learning process occurs through the interaction between the agent and the environment. Therefore, there is no dataset for learning as the other popular machine learning classes, i.e., supervised and unsupervised learning. The interaction between the agent and environment is achieved when the agent recognizes the current state within the environment, then it takes an action in order to transit to another state, see Figure 2. While the agent gets a positive (reward) or a negative (punishment) feedback when it reaches a good state or a bad state, respectively. Therefore, it is a slow trial-and-error process in which the agent learns from its experience. Although RL is a distinct machine learning from the supervised and the unsupervised learning paradigms; one could argue that it can be broadly viewed as a hybrid form of machine learning or semi-supervised approach as it takes a feature from supervised learning, which is the rewards feedback, while it takes another feature from unsupervised learning, which is no desired output.

There are three different RL methods [52]: Dynamic Programming, Monte Carlo, and Temporal Differences. The dynamic programming method requires a complete and accurate model of the environment; thus, it is for a model-based system in which the state transition is known. The Monte Carlo is a model-free method, i.e., the state transition is unknown, but is not suited for step by step incremental computation. In other words, it gives the feedback in the end of each experiment or game (an episode). The last method is the Temporal Difference (TD) that works with model-free applications and it is a fully incremental computation. In this study, the latter method is adopted as it is suitable for our project, which deals with a behavioristic system for a model-free application. The main components to design an RL model in any above-mentioned form are:

- States: the representation of perceived data. These representations include the final state, e.g., end of a game, or destination, e.g., final location in robotics. The number of states might be finite, or infinite based on the problem and the way of encoding them.
- Actions: the list of movement behaviors that need to be taken to change the state. The goal is to select the best action in a certain state that leads to a desirable state.
- Policy: the procedure of action selection at a certain state. It can be executed through a simple function or a lookup table, or it can be a stochastic function depending on the problem.
- Rewards: the immediate feedback after every transition. If the transition was good, then positive feedback (rewards) should be given to the agent. However, if the transition was bad, then negative feedback should be given to the agent, which needs to change the policy of selecting the action.
- State value: the collected rewards in the long term. If this is the value for a pair of (state-action), then it is called Q-value. The main goal of RL is to maximize this value to address the decision-making problem. It should be updated after every step in TD methods.
- An episode: is the end of every round of the problem. For example, the end of a game when the agent wins/loses is an episode or in robotics, when the robot achieves a specific task is an episode.

**Figure 3:**
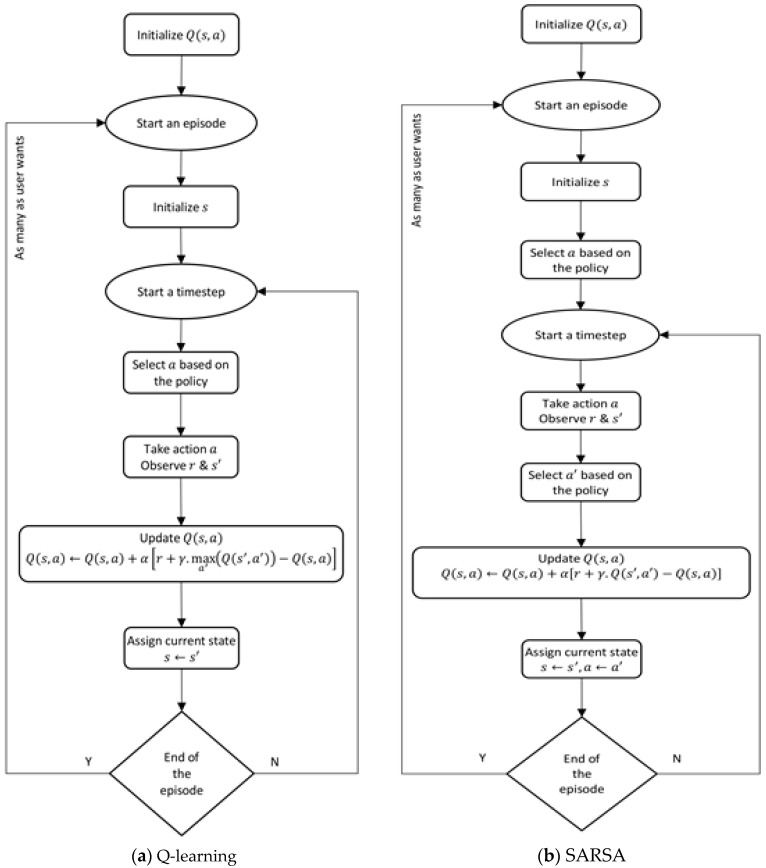
Temporal Difference algorithms of RL.

There are two popular algorithms of the TD method: Q-learning and SARSA (Sense-Act-Reward-Sense-Act). In both algorithms, the objective is to maximize the long-term reward Q(s,a), which represents the long-term reward for the combination of all states with all actions. The policy of selecting an action can be either exploiting or exploring. Exploiting movement is a 100% greed-move that selects the best-learned action with the highest value of Q(s,a). Exploring movement, on the other hand, is a ϵ-greedy-move that selects a random action. The difference between Q-learning and SARSA is in the way of updating the Q(s,a) in every time step, as shown in Figure 3. In the Q-learning algorithm, the maximum value of the future Q(s′,a′) will be used to update the current value of Q(s,a) even if the action a′ has not been selected for the next time step. For that reason, Q-learning is called an off-policy TD control. Whereas, the updating equation of the current value of Q(s,a) in the SARSA algorithm uses Q(s′,a′) where a′ should be the selected action for the next timestep, which is the reason this algorithm is called on-policy TD control. Therefore, they are similar when the policy of selecting the action is exploiting movement, i.e., 100% greedy move. There are several areas of RL applications including but not limited to games, e.g., backgammon or chess, inventor management, dynamic channels allocation, elevator scheduling, helicopter control, robotics, e.g., navigation, grasping, or Robocup soccer [53]. In this study, an appropriate reinforcement learning module was designed for obstacle avoidance as an adaptive behavior while exploring using limited sensors, i.e., two sonars.

## 3. Proposed System and Methodology

**Figure 4:**
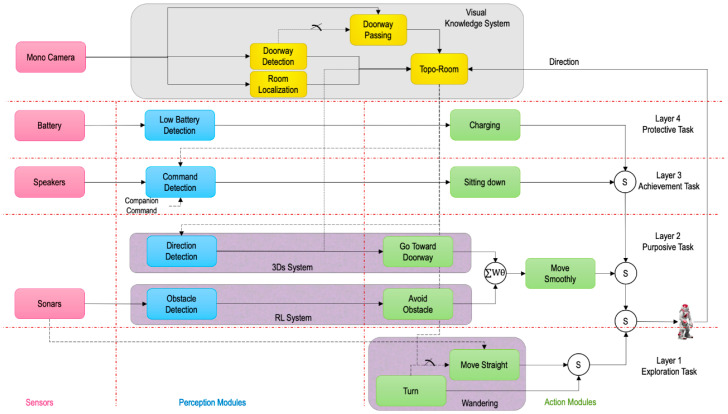
Learning-based behavioristic system for houses’ environments.

The proposed system is a hierarchical design inspired by the behavioristic paradigm of subsumption architecture augmented with a knowledge system. The behavioristic system is essentially multi-layered, whereby each layer is dedicated and responsible for a specific task—see Figure 4. In principle, the first layer (exploration task) is continuously active during the entire navigation task. The second layer (purposive task) takes the control and subsumes the exploration function. Similarly, whenever a higher layer is triggered, then the lower layers will be subsumed as explained in Section 2. However, the decision of the control layer does not only rely upon on the stimulus functions but also on the information from the knowledge system. The learning-based knowledge system is responsible for building a Directional Semantic Topological Map (DST-Map) that depends on the zone-based location and the related direction via a monocular camera. Most layers in the behavioristic system have bidirectional access to the knowledge system.

The detailed design with all layers, perception and action interconnected modules is shown in Figure 4. The first layer is always activated for exploring the environment and finding the purpose of the navigation task. It has only action modules with no perception modules: “Turn” and “Move Straight” behaviors. This implies that these action modules are not functions of any sensor’s data, therefore, this layer is always activated unless the higher layers subsume its control. The perception and action modules in the second layer are designed to control the robot to move towards the sub-goal, for instance the doorway, in a safe manner (purposive task). Therefore, this layer has two perception modules: the “Obstacle Detection” module, which is stimulated by sonar sensor, and the “Direction Detection” module, which is triggered by the knowledge system. Whereas, the action modules of this layer that control the robot are either “Go Toward Doorway”, “Avoid Obstacle”, or the weighted summation of both behaviors based on the perception modules outputs. Next, the achievement task in the third layer is responsible for making sure that the robot reaches the final goal assigned by a companion. Thus, the “Command Detection” module can be designed using the speaker to detect the companion’s command, such as “Come to the living room”, as well as comparing the commands with the current information in the knowledge system. If the robot reached the goal, then the navigation’s process will be ended by the action “Sitting down”. However, if the robot’s battery level drops to a certain level in any time during the navigation process, then the protective task in the fourth layer will be triggered and subsumes all other layers. Thus, the “Charging” action will take the control, which is basically in this work considered as a robot’s request to be charged. It can be made a more complicated module by designing an autonomous “Charging” module with more global information of the socket’s location or by local information of the socket’s detection in every room, which is out of the scope of this study.

The proposed system is designed and tested with few assumptions. First, the five different types of rooms are assigned for the CNN model with labelled images, in which the corridor is not one of the classes (see [37]). Second, we assume the practical predictions of classifying the room (see [38]) and detecting the doorway (see [54]) are always true positive within the navigation task. Third, we assume that the shape of all rooms is rectangular, and the connection between rooms is through one of the four directions (east:0°, west:180°, north:90°, south:−90°). Fourth, as the “command detection” cannot be tested with the simulator, the goal will be assigned by the user. In the following sub-sections, we explain the design of each module in more details.

### 3.1. Subsumption-Based System

The objective of this part is to design a collection of behaviors and interconnect them properly within four main layers, as follows:

#### 3.1.1. Layer 1: Exploration Task

**Figure 5:**
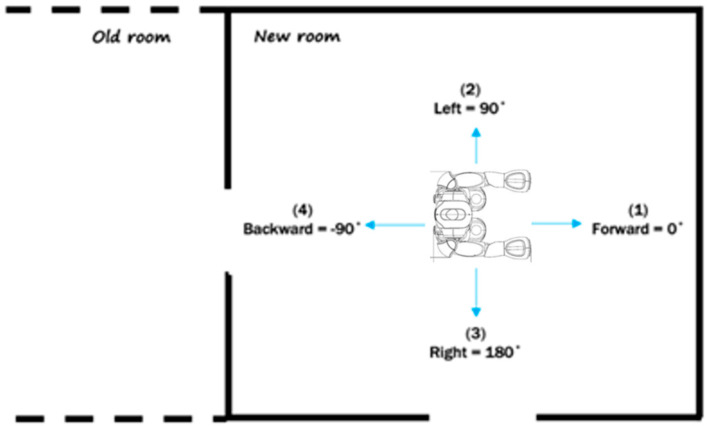
Illustration of robot’s direction while exploring.

Turn Module: this module consists of three predefined turning angles {90°, 180°, −90°} in an order that are related to all other possible directions in a room from any current direction. We assume all rooms are broadly a rectangular shape. The robot is supposed to detect the doorway in one of these directions, and the associated direction with the doorway will be passed to the knowledge system. The reason for this particular order is to minimize the number of turning. Let us assume that the robot enters a new room, which implies a doorway is behind the robot, as illustrated in Figure 5. The first direction that the robot tries to detect another doorway is the front, which is considered as 0° angle. If there is no doorway, then the robot selects the option of 90° (turn left). If there is still no doorway, then the robot turns to the other direction by applying 180° from the last direction. By now, the robot detects all three directions of this new room with only two moves. The last angle −90° (turning right) will be applied when there is no other doorway in the room and the robot is supposed to turn back to the previous doorway. The global direction will be passed to the knowledge system for the direction between nodes in the DST-Map.

Move-Straight Module: this module aims to change the scene in front of the robot by changing the position. It is triggered by either the “Turn” module in the same layer or the “Topo-Room” module in the knowledge system. “Turn” module triggers this module when the robot examines all directions but cannot detect a doorway. This is most likely due to the doorway being out of the robot’s view from its current position. Then, this module controls the robot to move straight through the best free space of the four directions based on the sonar values in order to change the view and improve the chance of detecting the doorway from a new spot. Therefore, the sonar values will always be passed to this module. The “Topo-Room” module triggers this module when the knowledge system gives a positive sign for passing the doorway, then it moves straight to make sure it is completely outside the previous room.

#### 3.1.2. Layer 2: Purposive Task (Obstacle Detection and Avoid Obstacles Modules: RL System Based on Sonars and Cautious Actions)

The key feature of this module is to design an adaptive behavior with a learning capability. The ultimate goal is to design an appropriate RL model (states, actions, and rewards) based on limited sensors on Nao, only two sonars. Each sonar provides a distance to an obstacle. We classify any distance into three classes {very close distance, close distance, far distance}—see Equation (1). Therefore, we can get nine different states, as shown in Table 1. As we can see, the best state is [2,2] where the robot has a good distance from obstacles from the front, whereas the worst is [0,0] where the robot is very close to obstacles from both sonars.

(1) $$S_{l}\&S_{r}=\{\begin{array}{c} 2,\;\;1.5\;m<far\;distance\leq 2\;m\\ 1,\;\;0.8\;m<close\;distance\leq 1.5\;m\\ 0,\;\;very\;close\;distance\leq 0.8\;m \end{array}.$$

The reward function is designed based on the above defined states. We divide the reward system into states rewards $R_{s}$ and transition rewards $R_{t}$, as shown in Equations (2) and (3), respectively. $R_{s}$ provide a positive or a negative reward based on the new state after taking a specific action. Whereas, $R_{t}$ is a measure of how good or bad the transition is; thus, it is calculated based on the difference of the old and new states. For example, if the robot was in state [0,1] and it moves to state [0,2], then the $R_{s}=-1$ in both states. However, since the transition is good, the robot gains positive reward for that transition $R_{t}=sum\left(\left[0,2\right]\right)-sum\left(\left[0,1\right]\right)=+1$. Then, the total reward will be the summation of the $R_{s}$ and $R_{t}$. Every episode is terminated either when the robot is able to roam for 500 s as maximum time for the positive termination, or when it falls down, which is associated with a final negative reward, i.e., r = −5. The maximum time of 500 s was selected based on several experiments, and it was found that it is a suitable time for an episode as the robot was able to explore most of the area during this time.

(2) $$R_{s}=\{\begin{array}{c} -2,\;\;if\;state=\left[0,0\right]\\ -1,\;\;if\;state\;has\;\left\{0\right\}\\ 0,\;\;if\;state=\left[1,1\right]\\ 1,\;\;if\;state=\left[2,1\right]or\left[1,2\right]\\ 2,\;\;if\;state=\left[2,2\right] \end{array},$$

(3) $$R_{t}=sum\left(state_{new}\right)-sum\left(state_{old}\right).$$

**Figure 6:**
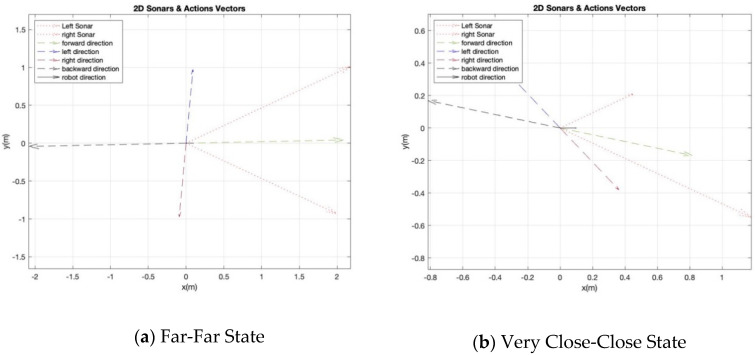
Action’s design of RL for an obstacle avoidance module.

The last important part of the RL system is the action function. The main four actions in the obstacle avoidance behavior are {Go Forward = GF, Turn Left = T L, Turn Right = TR, Turn Back = TB}. In order to avoid assigning predefined angles and distances for each action, we design an action function that takes into account the two sonars’ values to determine the distance and angle of each possible action. In other word, the exact values of distance and angle vary in every step for each action. This is achieved by calculating four different direction vectors, in which each one belongs to a specific action. Figure 6 shows different examples.

**Figure 7:**
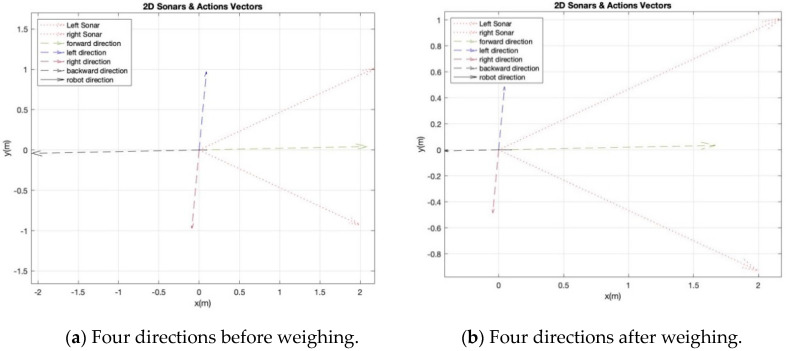
The impact of cautious action with RL system.

In order to avoid any dangerous action during the RL process, we suggest cautious actions by weighing each direction vector based on its current state. Let us assume that the robot is in the [2,2] state, which means the robot is far from obstacles from both sonars, as shown in the example of Figure 6a. If the robot selected the TB action, which has a large magnitude of distance, then it is better that this value has a low weight with this state as the robot does not know what obstacles are in the back. Therefore, each direction vector can be weighted by one of the three values $W=\left\{w_{1}=0.8,\;w_{2}=0.5,\;w_{3}=0.2\right\}$. These weights are for independent actions; thus, they do not have to be a summation of 1. The small weight is only to avoid applying the full action when the robot is exploring in a dangerous scenario, such as turning back where the robot has no clue what obstacles are behind. The cautious action process and the associated weights are shown in Algorithm 1 and Table 2 respectively. While the effect of weights on the action vectors is shown in Figure 7.
**Algorithm 1.** Cautious Action Process of RL Model.
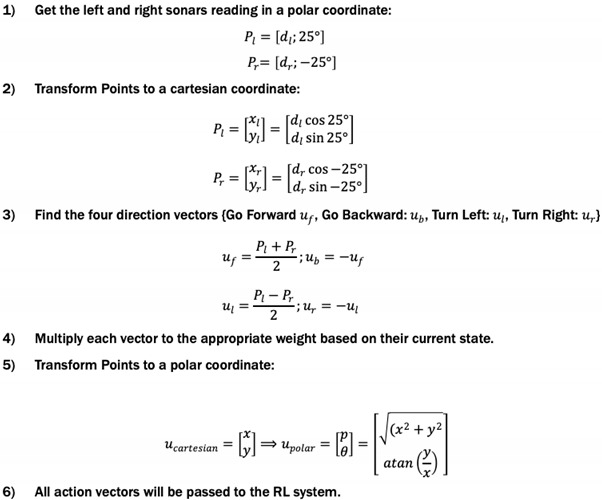


Direction Detection and Go Towards Doorway Modules: the “Direction Detection” perception module is triggered by the provided information from the knowledge system. If the doorway detection attribute in “Topo-Room” map is positive, then this module will be activated for running the system explained in [54]. As this system is able to detect if the deepest information is not related to the doorway, then the doorway detecting within the knowledge system will be updated. However, if the deepest information is related to the doorway, the calculated angle will be passed to the “Go Toward Doorway” action module to turn and translate the robot toward the doorway.

Move Smoothly Module: in cases where the robot should move to the detected direction of the doorway where there is an obstacle in the way, designing a smooth move is important. This action module is the weighted summation of the output from the preceding action modules, i.e., “Avoid Obstacle” and “Go Toward Doorway”. Let us call the action from “Avoid Obstacle”, “Go Toward Doorway”, and “Move Smooth” as $a_{o}$, $a_{g}$ and $a_{s}$, respectively, in which each action consists of angle and translation values. When the robot is very close to the obstacle, then the “Avoid Obstacle” action takes the full or the higher control. Whereas, if there is no obstacle or they are far enough, then the “Go Toward Doorway” action takes the higher weight. This is executed by applying the following weighted summation function:

$$a_{s}=w*a_{o}+\left(1-w\right)*a_{g}.$$

#### 3.1.3. Layers 3 and 4: Achievement and Protective Tasks

Modules in these two layers are designed using the pre-defined functions from the Naoqi API [55] in order to terminate the navigation process. The achievement task is to end the navigation process via following a voice command made by a companion using Nao’s speaker. The “Command Detection” perception module can be designed by detecting one of the five room classes from room classification component, as presented in [37]. By comparing the command and the information in the knowledge system, the “Sitting Down” action module will be run as an indication of completing the navigation process. Similarly, the protective task is important to keep the robot protected from falling if the battery is out of charge. The battery level is checked all the time during the process of navigation. If the level is low, e.g., less than 20%, then the system applies the “Charging” action module. As this function is not mainly a part of navigation in this project, we keep this module simple by applying a request function that the robot asks to be charged.

### 3.2. Knowledge-Based System

The behavior-based approach for the navigation system was inspired by the concept of behaviorism, which speculates that behaviors are triggered by the environment. In contrast, having mental representations that play a causal role in behaviors was the assumption stated by cognitive psychology [20]. Accordingly, we modify the subsumption-based system by integrating a knowledge-based system that is a crucial part in the learning phase. We adopt the topological-based mapping approach for achieving the knowledge part of the system. Topological map [56] is a graphical representation of the environment that consists of nodes and edges. Nodes represent different places while edges are the connection between relative positions of these places.

Room Localization and Doorway Detection Modules: (CNN-Based Models): the knowledge-based system aims to create a Directional Semantic Topological Map via designing a module called “Topo-Room”, which will be explained below. Therefore, the system has to begin with “Room Localization” and “Doorway Detection” perception modules that successfully designed and tested, using the CNN model as explained in detail in [37,38,54], respectively. “Room Localization” provides the semantic feature, whereas “Doorway Detection” provides the directional feature to the topological map.

SRIN dataset [38] was employed to train a CNN-based model via transfer learning process, in which extracting features part was executed through frozen layers of VGG16 and the classifier part was executed through fully connected (FC) network. The two modules were programmed in python using Keras API [57], and the training and validation process was completed offline through the Graham cluster provided by Compute Canada Database [58].

**Figure 8:**
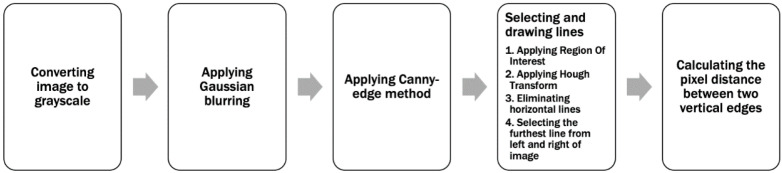
Doorway’s edges detection for “Passing Doorway” module.

Doorway Passing Module: (Canny Edge Detection and Hough Transform): when the robot identifies the room’s type and detects the doorway, then there is no need to keep applying them again while the robot wanders in the same room. Thus, this is the key role of integrating a knowledge system with the behavior-based navigation system. When the robot moves to a new room, then it needs to capture a new image for adding new knowledge to the “Topo-Room”. Therefore, we design a “Passing Doorway” perception module based on the Canny edge detection [59] and the Hough transform [60], as an indicator that the robot left the current room and arrived at a new room. The key idea is to extract the two edges of the doorway from a 2D image towards the doorway. The distance in pixels between the two edges will be increased while the robot gets closer to the door. When the two edges are out of the robot’s view, then it is most likely that there are new edges that will be detected with a smaller distance than the previous one, as will be shown in Section 4.1.2. Figure 8 shows the practical process of getting doorway edges.

Canny edge detection is a popular method that can be applied on a smoothed gray image by a Gaussian filter. The output of this stage is a set of points within the image. For that reason, applying the Hough transform is important in order to extract lines. For the purpose of this project, we extracted only the vertical lines with a specific threshold within a certain region of interest (ROI). We assumed that there are no objects with vertical lines, such as a shelf, besides the doorway. Therefore, we selected the furthest line from left and right of the image as the doorway edges.

**Figure 9:**
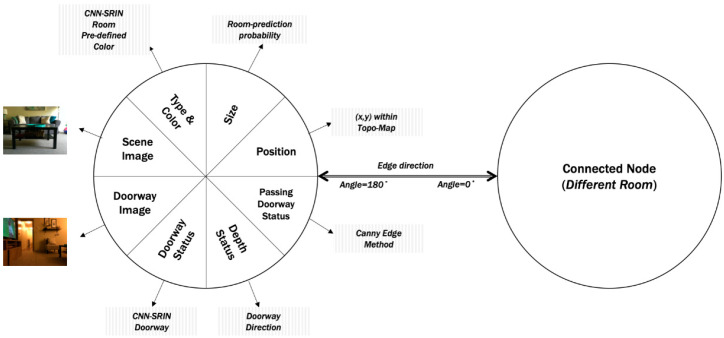
Main attributes or information within each node in the TDS-Map.

Topo-Room Module: (A Directional Semantic Topological Map): the “Topo-Room” module is a Directional Semantic Topological Map (DST-Map). It is semantic as each node is associated with a specific class of room that is provided by the “Room Localization” perception module. In addition, “Topo-Room” is directional because the relative position is based on the four directions {East, West, North, South} that can be extracted by “Doorway Detection” perception module and the predefined angles in the first layer (exploration task). The objective of this module is to build an abstract map for the house environment. The abstract map is a high-level representation that saves the connection between rooms. Thus, the created map is a collection of nodes that represent rooms, and these nodes are connected by edges that represent the direction towards the doorway. There are eight attributes associated with each node, as shown in Figure 9. They are as follow:

- The first two attributes are the type with a predefined color and the size. They are extracted from the “Room Localization” module, in which the type is the prediction of the room class and the size is the prediction’s probability.
- The node’s position, i.e., the third attribute, is assigned based on one of the four directions (east:0°, west:180°, north:90°, south:−90°) as the key is to find the relation direction between nodes. Thus, we assume that all nodes’ positions depend on the position of the first node. In other words, we assume that the first node for the first classified room is positioned in the (0,0) of the map, and the direction of its doorway is always in the east:0°. Then, the position of the next room depends on the position of the previous room and the global direction of the doorway by applying these two equations:
  $$next_{node_{x}}=pre_{node_{x}}+\cos\left(pre_{door_{angle}}\right),$$
  $$next_{node_{y}}=pre_{node_{y}}+\sin\left(pre_{door_{angle}}\right).$$
- Each node is associated with a saved scene image as the fourth attribute for future needs, such as appearance association or matching.
- The other attributes are related to the doorway within the room, which are extracted from the “Door Detection” perception module. Doorway status gives information about doorway detection as positive or negative. If it is positive, then the image will be saved as the sixth attribute, i.e., doorway image. The seventh attribute is for depth status, which gives information about the execution of “Doorway Direction”. If the module is executed and calculated the depth and direction toward the doorway, then its status is positive. Finally, the attribute of passing doorway status gives positive or negative information based on the “Passing Doorway” module, while it saves the distance between two edges every time it is needed. If its status is positive, then the process of gaining new information and creating a new node will start again.

**Figure 10:**
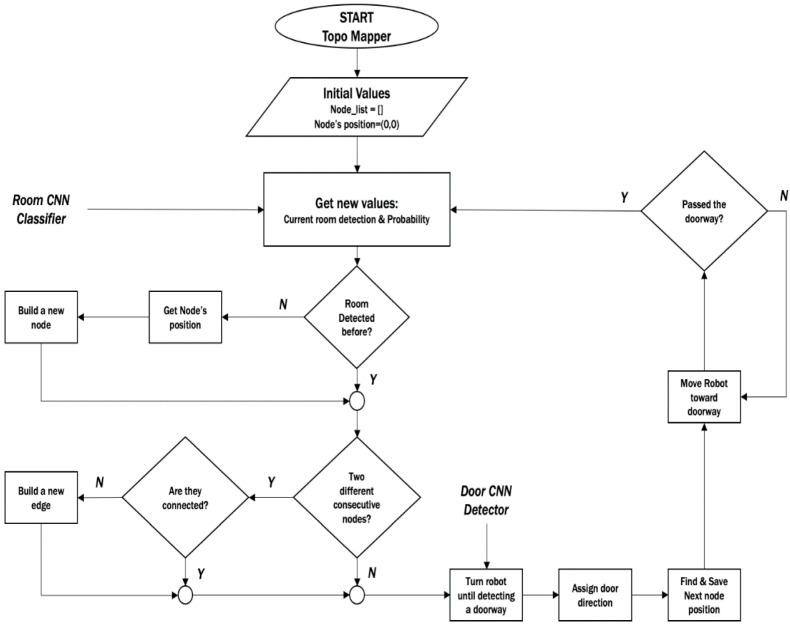
Flowchart of creating and updating the Directional Semantic Topological Map (DST-Map).

**Figure 11:**
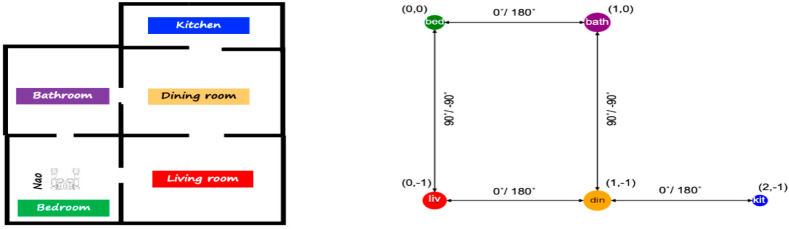
An illustration of the expected DTS-Map (**right**) for a simple layout (**left**).

The important attribute related to the edges is a bidirectional angle from the four directions between two classified rooms. The flowchart in Figure 10 shows the map building process of the “Topo-Room” module within the knowledge-based system. The expected DST-Map is an abstract map that contains nodes of rooms with their positions in the map space, and the edges between nodes that show the angular relation between two relative nodes, as shown in the illustrated example of Figure 11.

### 3.3. Implementation Setup

**Figure 12:**
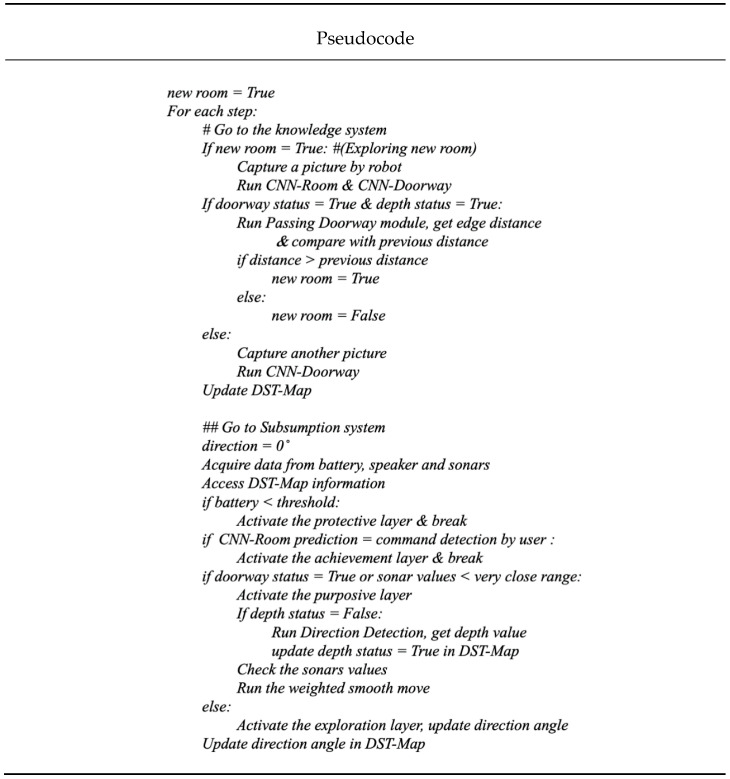
Pseudocode of implementing the overall system sequentially.

All modules were programmed by python language using a Laptop with a 64-bit Linux operating system with 8GB RAM. It has a Graphic Process Unit of Quadro K620M with 2GB total memory. As the system was implemented on a small memory size GPU, all modules were executed in a sequential manner as Webots takes most of the GPU size during the process. Therefore, Figure 12 shows the pseudocode of implementing all modules together within the system.

## 4. Experiments and Results

**Figure 13:**
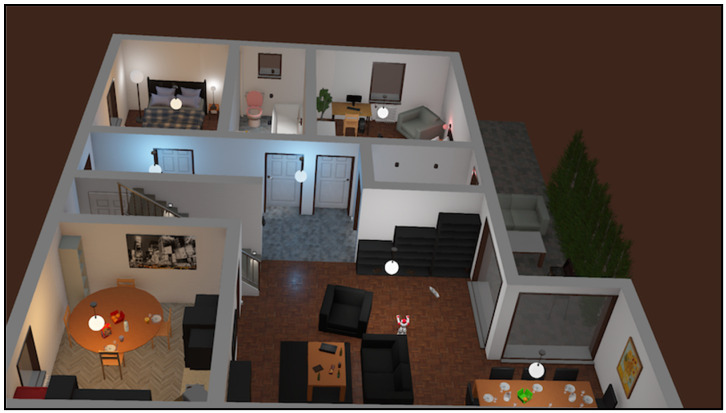
An apartment virtual model with Nao robot using the Webots simulator.

We have applied an apartment model with Nao humanoid robot using the Webots simulator [36] for executing all virtual-time experiments, see Figure 13.

### 4.1. Evaluation of Individual Modules

We adopted our previous work in [38,54] for the “Room Localization” and “Doorway Detection” modules, respectively, within the knowledge system, and the proposed system in [54] for the “Direction Detection” within the layer of the purposive task. The evaluation of the other important modules is shown in this section.

#### 4.1.1. Evaluating RL System for Obstacle Avoidance Module

All experiments have been executed within the virtual environment with a learning rate α=0.2, and a discount factor γ=0.8. In order to ensure a good learning process, a ϵ-greedy was added to the RL process—resultingly, the robot will not get stuck in certain areas and it can face all states’ situations as well as learn the best associated behavior. We suggest a gradual ascent ϵ-greedy for every 10-time steps in order to make sure that the robot keeps exploring in different areas rather than only exploring in the beginning of RL training process. Every step is considered as an action that is taken by the robot during the process. A gradual ascent ϵ-greedy combines the exploring and exploiting movements in every episode. So, we started with 60% of ϵ-greedy in the first 10-time steps in which the robot starts with exploring movements in the first four steps. Then, the ϵ-greedy is increased to 70% in which the robot explores in the first three steps of the second 10-time steps. Therefore, the robot increases the exploiting movement and decreases the exploring movement every 10-time steps until reaching 50-time steps when the robot moves while fully exploiting based on what has been learned during the RL process. Since the objective of this experiment is that the robot keeps avoiding obstacles as long as it can, then time of wandering is the key factor of evaluating the performance of this experiment. Each episode is ended either when the time of wandering reached 500 s or when Nao falls down. The process of RL is terminated when Nao is able to wander without falling for three consecutive episodes. Thus, Nao is able to move about 1500 s, i.e., over 25 min.

**Figure 14:**
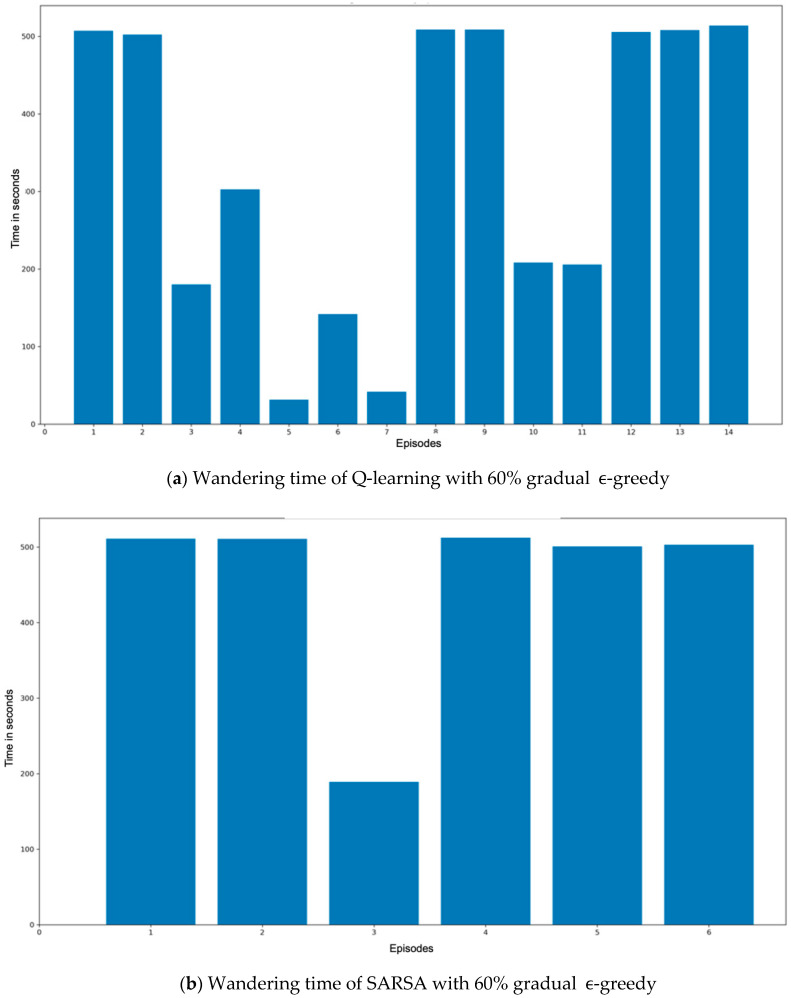
RL training results for both methods: (**a**) Q-learning and (**b**) SARSA.

Figure 14 shows the RL results by presenting the wandering time in every episode for both Q-learning and State–action–reward–state–action (SARSA) methods with gradual ϵ-greedy. Nao was able to wander around the living room for three consecutive episodes without colliding into obstacles after 14 episodes using the Q-learning method, as shown in Figure 14a, while it learned much faster using the SARSA method, as shown in Figure 14b. Therefore, the trained model of SARSA was adopted for the overall system’s evaluation.

#### 4.1.2. Evaluating Doorway Edges for “Passing Doorway” Module

**Figure 15:**
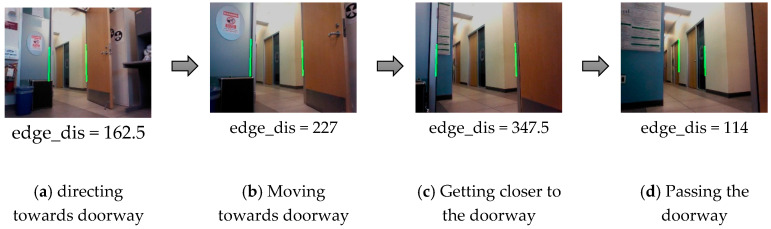
Doorway edges detection for the “Passing Doorway” module.

This module was tested with a simple real-time experiment with the Nao robot to evaluate the concept of passing the doorway based on detecting edges. Figure 15 shows a sequence of images while Nao moves toward the doorway in the AISL lab at SFU. As we can see, the distance between the two detected edges of the doorway is increasing when the robot gets closer to the door, as shown in Figure 15a–c. Whereas the distance becomes smaller when the robot passed the doorway, as shown in Figure 15d.

### 4.2. Evaluation of the Overall System

Several experiments with different scenarios are presented below for a virtual evaluation of the overall system using a Webots model. The objective of these scenarios is to show that the robot is able to move between two different rooms—specifically the kitchen and the living room, safely. Thus, it can maintain information of two connected rooms within the knowledge system, i.e., two connected nodes with an edge. This can be extended for exploring other rooms. Therefore, the results will not show the mapping part. All results for all scenarios are presented in two parts: (a) the actions’ decision making based on the behavior and knowledge systems in every step, and (b) the doorway perception’s output for both depth and edge detecting during the exploration process.

#### 4.2.1. Scenario 1: Moving between Two Rooms with No Obstacles

**Figure 16:**
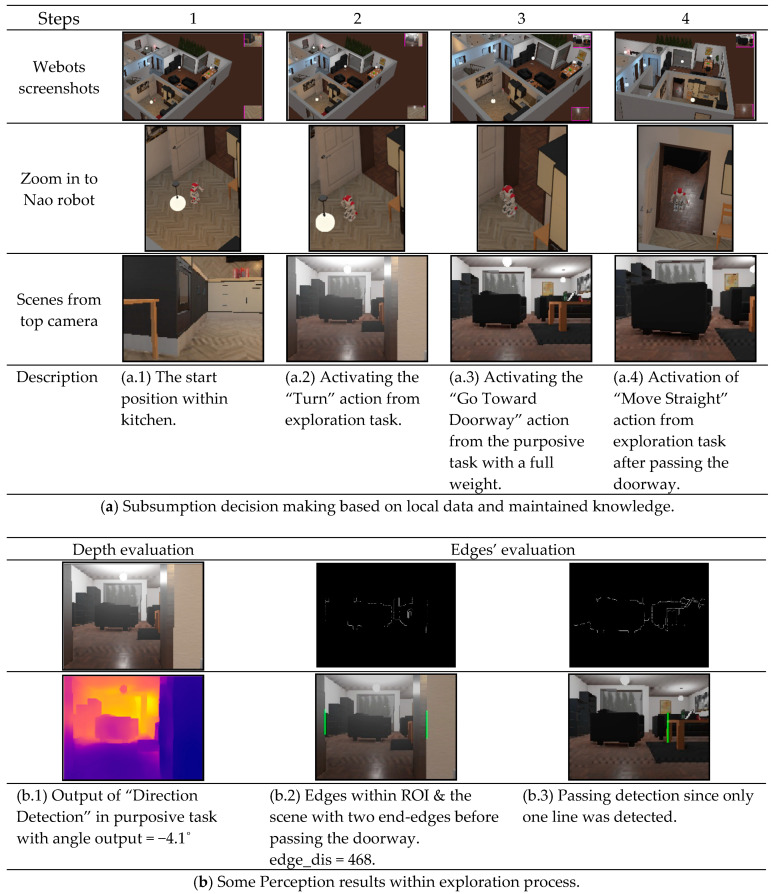
Evaluating the overall system in Scenario 1.

The demonstration of this scenario is to test the robot’s ability to move from the kitchen to the living room with no obstacles in the way. We try to test the robot’s ability to recognize the current room and find the right direction of the doorway to connect the two subsequent rooms within the map. Additionally, the robot is tested to decide when it passed the room to start a new room recognition and update the map. The detailed perceptions and actions of the system for this scenario are shown in Figure 16. The robot started from a position close to the doorway, which is in the left side of robot, in the kitchen while there were no other obstacles. The robot was able to predict the kitchen with a highest probability of 79.4% compared to other classes, while the status of doorway detection was “no-door”. Therefore, the first layer (exploration) was activated, and the robot turned by 90˚ to the left. Now, the robot was able to detect the doorway and change its status in the DST-Map, thus the “Direction Detection” was activated and calculated a small angle between the robot and the doorway. During the experiments, we considered any calculated angles from depth information within the range of [−10°,10°] as small values, so the robot did not need to turn that small values. Hence, the robot almost directed to the doorway, the “Passing-Doorway” module detect the doorway edges and calculated the distance. After that, the second layer (purposive) was activated, in which the “Go Toward Doorway” took all the weight of the smooth movement. Then, the system compared the new edge distance, which was zero as only one edge was detected, with the previous saved distance in the knowledge system. Since it was smaller, the status of the “Passing Doorway” attribute within the DST-Map was changed, and the “Move Straight” action in the first layer (exploration) was activated to make sure a full pass to the new room was made. Subsequently, gaining new knowledge started again by classifying the new room as a living room with 93.7%.

#### 4.2.2. Scenario 2: Moving between Two Rooms with a Different Direction

**Figure 17:**
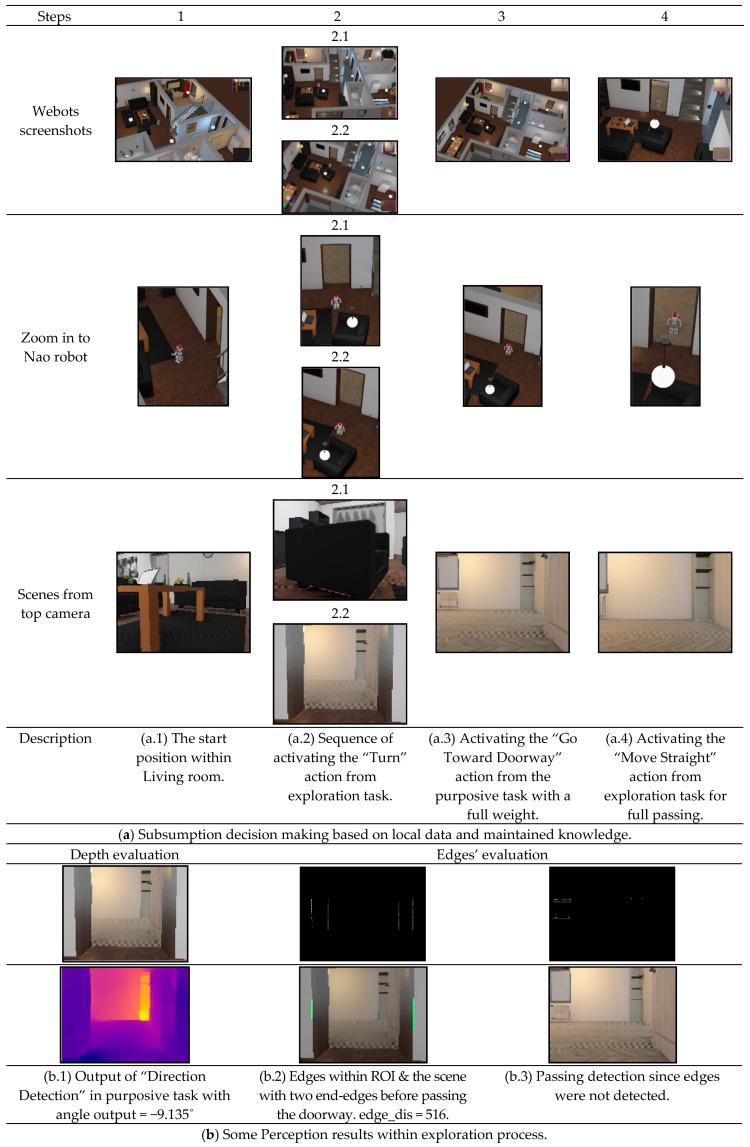
Evaluating the overall system in Scenario 2.

The difference in this scenario is that the direction of the doorway is in the right side of the robot. The advantage of angle directions order in the exploration task can be showed in this scenario. Similar to the first scenario, the robot is tested to recognize current room and find the right direction of the doorway as well as being tested to pass the room and start a new room recognition while updating the map information. We tried in this scenario to start the experiment from the opposite room, i.e., the living room, as shown in the detailed results of Figure 17. The first obtained information by the knowledge system was classifying the room correctly with a 96.4% prediction’s probability. The exploration task was activated twice by turning the robot by 90° and 180° consecutively until detecting the doorway. Once the doorway was detected and its corresponding attribute was updated in the knowledge system, the depth information was calculated with a small value. Consequently, the doorway edges were found with a large distance between edges, which indicates that the robot is very close to the door and ready to pass it. As there were no obstacles, the “Go Toward Doorway” action in the purposive task was activated and took the all weight of “Smooth Move”. Once the “Passing Doorway” status updated in the knowledge system, the robot started gaining a new knowledge and building a new node of kitchen room with 83.3% prediction’s probability.

#### 4.2.3. Scenario 3: Moving between Two Rooms with Obstacles and a Final Goal

**Figure 18:**
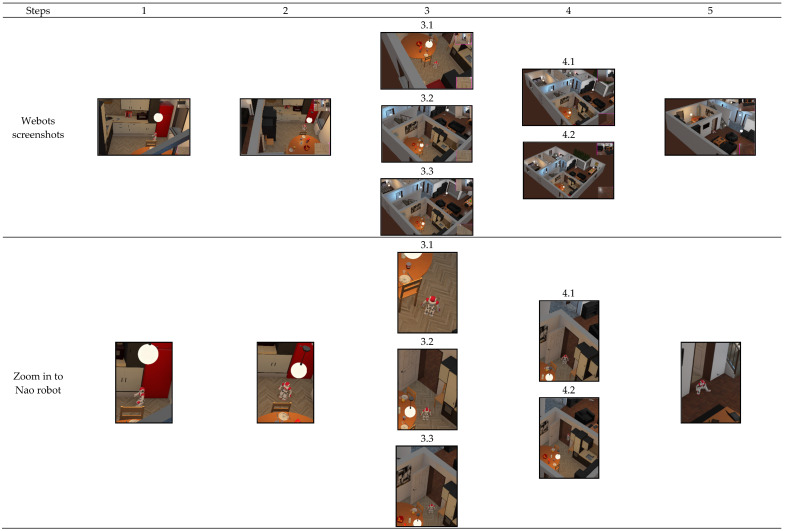

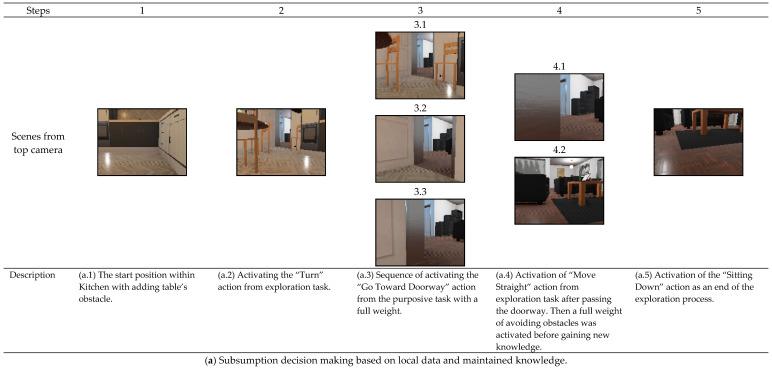

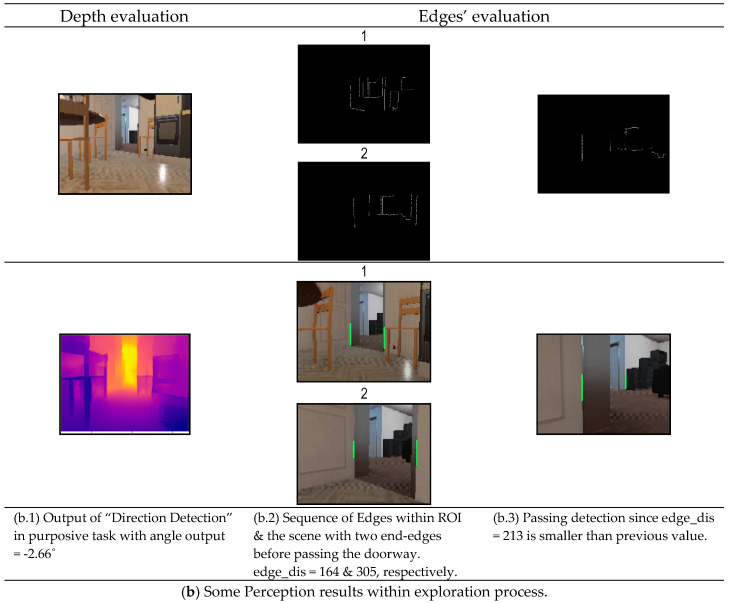
Evaluating the overall system in Scenario 3.

Including the objectives from last scenarios, the objective of this scenario is also to test obstacles avoidance and moving smoothly towards the doorway, as shown in Figure 18, by adding extra objects around the starting position. Additionally, the final target is assigned from the beginning to test the performance of the achievement task. The robot started from the kitchen and its goal to go to the living room as a final targeted room. We designed the system to activate the weighted “Avoiding Obstacle” action if one of the sonars is less than 1 m within the “Move Smoothly” action, while a full weighted “Avoiding Obstacle” if one of the sonars is less than 0.4 m. So, the robot started by gaining information about 96.3% prediction of the kitchen. Then, the exploration task was activated, and the robot turned by 90˚ to the left direction. Now, many perceptual modules were activated sequentially to acquire doorway-related information, i.e., doorway detection, doorway direction, and doorway edges as explained in the previous scenarios. We noticed that during the robot’s actions from the purposive task, the sonar readings were no less than 1 m. Therefore, the robot kept moving toward the doorway. The doorway edges were detected, and their distances were calculated three times consecutively. Once the edges’ distance became smaller than the previous save distance, the status of “Passing Doorway” was updated to positive. Now, instead of activating “Move Straight” action from the exploration task for a full pass through the doorway, the system activated the “Avoid Obstacle” action within the purposive task via RL since the sonars values were (left = 0.38 m and right = 2.5 m). When the robot completely moved away from the obstacle, which was the door edges in this experiment, it gained new information, starting by classifying the new room as a living room with 97.3%. Since the classified room matched the target room, the “Sitting Down” action was activated as an indication of ending the process.

## 5. Discussion

The proposed system is flexible as each module can be designed, tested, and modified individually and within the overall system. The virtual experiments showed promising results, in which the system can be adopted and modified for any social robot with limited sensors for domestic applications. However, we focus in this section more on some of the limitations or areas for improving the results. The RL for “Avoiding obstacle” worked very well during the training stage within the area of the living room. However, this room has a spacious area, thus, the RL can be applied in another room for more testing and improving the learning process before adopting the final model within the system. Additionally, the depth images with their calculated angles from Scenarios 1 and 2 were not the best expected angles to keep the robot’s direction exactly in the middle of the doorway. This might be due to the inaccuracies in the simulator’s view, as the reported results from [54] were better and more accurate. Thus, we ignored the small values in the virtual experiments and let the robot move straight towards the doorway. In addition, adding a class of corridors for the room classification project is important for future work in order to extend the exploration process between more rooms. Thus, building the topological map will be more meaningful.

Although extensive simulation runs with Webots were conducted, we suggest that the real-time experiments with NAO will produce similar results as Webots since this software uses the same NAOqi API as the actual NAO humanoid robot. Additionally, we expect that the process with real-time experiments will be much faster than the virtual experiments as Webots takes most of the GPU memory during the navigation process. This will be avoided in the real-time implementation.

Therefore, the proposed system is an alternative solution for addressing localization and mapping sequentially for indoor environment, specifically homes. We coined the term Sequential Localization and Mapping (SeqLAM), and provided a qualitative comparison to the widely popular probabilistic Simultaneous Localization and Mapping (SLAM) algorithm, as shown in Table 3. The only prior information that SeqLAM requires is that the environment to be explored (home) has five classes (bathroom, bedroom, dining room, kitchen, and living room). Humans have this prior information when they go to a new “home”. They know that the place has a kitchen and a dining room, but they do not know their spatial relation. They discover this only after exploring the environment. SeqLAM can be designed for any indoor environment (such as a hospital, a school, etc.) provided that the prior knowledge of different classes is available.

## 6. Conclusions

This paper presented a navigation strategy for social robots with limited sensors within apartments’ environments. The design combined a subsumption-based system and knowledge-based system. The subsumption system consisted of a collection of behaviors arranged in layers, in which each layer was responsible for a specific task and became activated based on the sensor data. Whereas, the knowledge system consisted of several visual-learning modules to gain information about the environment to build a high-level meaningful map as well as to access all layers in subsumption and trigger the appropriate action. Some modules were evaluated individually in virtual or real-time implementation with the Nao robot. For example, an RL model was designed properly with two different approaches, Q-learning and SARSA, for obstacle avoidance as an adaptive behavior using only two sonars, and the model was evaluated virtually by observing the time of exploration and number of epochs. The model with the SARSA approach learned faster than the Q-learning. “Passing Doorway” was the other module that was evaluated individually in this paper. It was tested in a simple real-time experiment to evaluate the concept and to be adopted with the overall system. On the other hand, the overall system was tested virtually using Webots simulator with different scenarios. Although there were some restrictions and assumptions, the performance of all scenarios showed promising practical results.

## Figures and Tables

**Table 1:** RL model states for avoiding obstacle behavior.

States	$S_{l}$	$S_{r}$
1	0	0
2	0	1
3	0	2
4	1	0
5	1	1
6	1	2
7	2	0
8	2	1
9	2	2

**Table 2:** Associated weights with states.

Direction Weights	GF	TL	TR	TB
S1=[0 0]	0.2	0.2	0.2	0.8
S1=[0 1]	0.2	0.2	0.5	0.2
S1=[0 2]	0.2	0.2	0.8	0.2
S1=[1 0]	0.2	0.5	0.2	0.2
S1=[1 1]	0.5	0.5	0.5	0.5
S1=[1 2]	0.5	0.5	0.5	0.2
S1=[2 0]	0.2	0.2	0.8	0.2
S1=[2 1]	0.5	0.8	0.5	0.2
S1=[2 2]	0.8	0.5	0.5	0.2

**Table 3:** Qualitative comparison between classic SLAM and SeqLAM.

Features	SLAM	SeqLAM
Philosophy	Probabilistic	Behavioristic
A priori knowledge	Assigning Landmarks	Zone based (in homes)
Update information	Incremental robot’s pose and map	Identifying zones, then generating a map sequentially
Map	Accurate	Sketch/spatial relationship
Pose	Accurate	Not applicable
Dynamic settings	Numerous challenges	Moderate
Computation load	High	Moderate
Comparison to human reasoning	Not intuitive	Intuitive
Generality	Outdoor/Indoor	Indoor
Data Association	Significant challenge	Can be incorporated via image matching
Human intervention	Robot is mostly driven manually (except for autonomous exploration)	Autonomous
Application	Universal (but needs to be tailor made)	Only homes (can be tailor made for other indoor settings, e.g., hospitals)
